# The Role of Mitochondria in Mood Disorders: From Physiology to Pathophysiology and to Treatment

**DOI:** 10.3389/fpsyt.2021.546801

**Published:** 2021-07-06

**Authors:** Anna Giménez-Palomo, Seetal Dodd, Gerard Anmella, Andre F. Carvalho, Giselli Scaini, Joao Quevedo, Isabella Pacchiarotti, Eduard Vieta, Michael Berk

**Affiliations:** ^1^Bipolar and Depressives Disorders Unit, Hospital Clínic, University of Barcelona, Institut d'Investigacions Biomèdiques August Pi i Sunyer (IDIBAPS), Mental Health Research Networking Center (CIBERSAM), Madrid, Spain; ^2^Deakin University, The Institute for Mental and Physical Health and Clinical Translation, School of Medicine, Barwon Health, Geelong, VIC, Australia; ^3^Department of Psychiatry, Centre for Youth Mental Health, The University of Melbourne, Melbourne, VIC, Australia; ^4^Centre for Addiction and Mental Health, Toronto, ON, Canada; ^5^Department of Psychiatry, University of Toronto, Toronto, ON, Canada; ^6^Translational Psychiatry Program, Faillace Department of Psychiatry and Behavioral Sciences, McGovern Medical School, The University of Texas Health Science Center at Houston, Houston, TX, United States; ^7^Neuroscience Graduate Program, The University of Texas MD Anderson Cancer Center UTHealth Graduate School of Biomedical Sciences, Houston, TX, United States; ^8^Translational Psychiatry Laboratory, Graduate Program in Health Sciences, University of Southern Santa Catarina, Criciúma, Brazil; ^9^Center of Excellence in Mood Disorders, Faillace Department of Psychiatry and Behavioral Sciences, McGovern Medical School, The University of Texas Health Science Center at Houston, Houston, TX, United States; ^10^School of Medicine, The Institute for Mental and Physical Health and Clinical Translation, Deakin University, Barwon Health, Geelong, VIC, Australia; ^11^Orygen, The National Centre of Excellence in Youth Mental Health, Parkville, VIC, Australia; ^12^Centre for Youth Mental Health, Florey Institute for Neuroscience and Mental Health and the Department of Psychiatry, The University of Melbourne, Melbourne, VIC, Australia

**Keywords:** mitochondrial dysfunction, mood disorders, bipolar disorder, major depressive disorder, novel therapies

## Abstract

Mitochondria are cellular organelles involved in several biological processes, especially in energy production. Several studies have found a relationship between mitochondrial dysfunction and mood disorders, such as major depressive disorder and bipolar disorder. Impairments in energy production are found in these disorders together with higher levels of oxidative stress. Recently, many agents capable of enhancing antioxidant defenses or mitochondrial functioning have been studied for the treatment of mood disorders as adjuvant therapy to current pharmacological treatments. A better knowledge of mitochondrial physiology and pathophysiology might allow the identification of new therapeutic targets and the development and study of novel effective therapies to treat these specific mitochondrial impairments. This could be especially beneficial for treatment-resistant patients. In this article, we provide a focused narrative review of the currently available evidence supporting the involvement of mitochondrial dysfunction in mood disorders, the effects of current therapies on mitochondrial functions, and novel targeted therapies acting on mitochondrial pathways that might be useful for the treatment of mood disorders.

## Introduction

Mitochondria are cellular organelles known to be involved in diverse biological processes, such as adenosine triphosphate (ATP) production, metabolism of reactive oxygen species (ROS), calcium (Ca^2+^) homeostasis, cell death and survival (1), as well as in synaptic plasticity. Mitochondria are abundant in neuronal dendrites and synaptic terminals. In the brain, which uses high amounts of ATP and does not have the ability to store it (2), their activity is crucial for the modulation of neuronal activity, short- and long-term neuronal plasticity, cellular resilience, and behavioral adaptations, mainly through actions on long-term potentiation (3–6). Moreover, several lines of evidence suggest roles for mitochondria in supporting the different bioenergetic requirements of highly proliferative neural stem cells and postmitotic neurons (7). In this respect, the adaptation of the energy supply to the energy demand and mitochondrial health is central to cellular homeostasis, and appropriate neuronal function (8–10).

Mitochondrial dysfunction is considered a multifactorial phenomenon since it may have multiple causes and affects numerous neurobiological processes, altering synapsis and enhancing apoptosis, which could play a role in the potentially progressive long-term course of some psychiatric disorders (1). Several studies have focused on the presence of impaired energy metabolism in patients with mood disorders, which indicates that mitochondrial dysfunction may play an important role in various aspects of these conditions (2). In summary, the “mitochondrial hypothesis” suggests that mood disorders are triggered, in part, by mitochondrial dysfunction, which can be intimately linked to a wide range of processes associated with treatment outcomes, disease progression, and severity (11, 12). Moreover, mitochondrial dysfunction could pre-dispose vulnerable individuals to these disorders and lastly, be an important target for current and novel potential therapies for mood disorders (2).

Every cell depends on energy production from mitochondria, with much higher demand in neurons, especially in gray matter, which has a high number of synapses and mitochondria (13–15). Besides energy production, mitochondria are sources of cellular growth substrates and play crucial roles in oxidative/nitrosative stress, cell resilience, and death pathways (3, 16, 17).

Mitochondria are the only organelles in the cell that contain their own DNA, called mitochondrial DNA (mtDNA), which contains 37 genes that encode 13 proteins, 22 tRNA, and 2 rRNAs. These genes encode 13 protein subunits of the electron transport chain (ETC). Genes from nuclear DNA (nDNA) code the rest of the mitochondrial proteins (15) and play a role in the regulation of mitochondrial function. In contrast to nDNA, mtDNA is vulnerable to DNA damage due to constant exposure to reactive oxygen species (ROS) and at times insufficient DNA repair mechanisms (18). Moreover, a number of proteomic studies have been conducted to decipher the mitochondrial proteome. Several mitochondrial databases that list the number of mitochondrial proteins are available nowadays (19).

**Figure 1:**
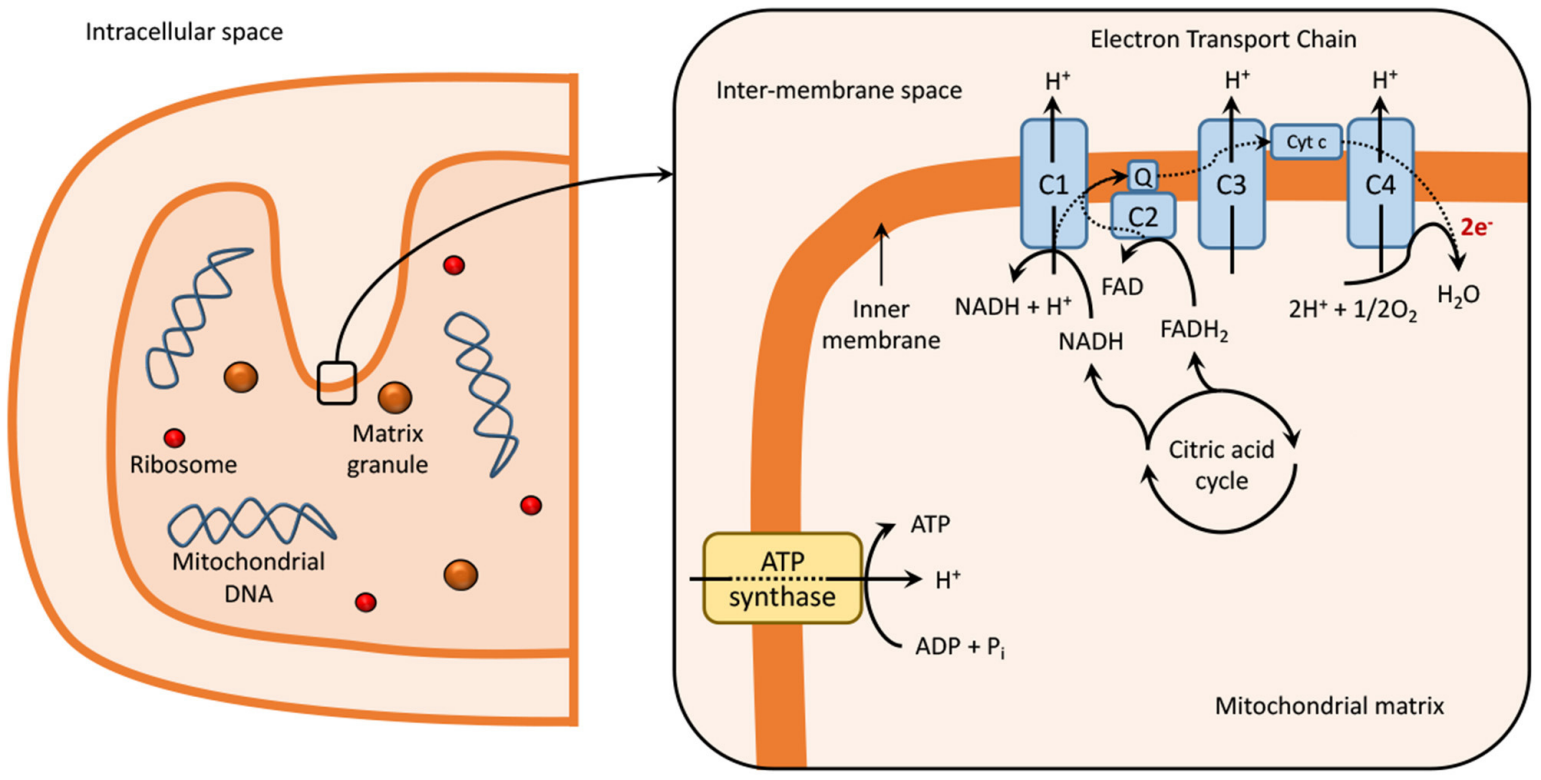
Mitochondria at normal physiological conditions. Dashed lines represent electron transport in the electron transport chain reaction (ETC). The ETC is localized within the inner mitochondrial membrane or cristae of the mitochondria (21–23) and is composed of five multimeric protein complexes (I-IV and ATP-synthase or complex V) that are responsible for ATP production by oxidative phosphorylation. Complex I, or nicotinamide adenine dinucleotide (NAD+), and/or complex II (succinate dehydrogenase), begin the process of oxidative phosphorylation by catalyzing the transfer of electrons from NADH and FADH_2_, respectively, to coenzyme Q (or ubiquinone). The transfer of electrons is serially conducted through complex III (ubiquinol: cytochrome c oxidoreductase), cytochrome c and complex IV (cytochrome c oxidase), to the terminal acceptor, generating an electrochemical proton gradient that enhances ATP production in complex V *via* oxidative phosphorylation (1, 24, 25). During this process, single-electrons can escape and produce a single-electron reduction of O_2_, forming superoxides and other ROS (24, 26, 27). Impaired functioning of ETC can result in excessive ROS production, which leads to the damage of DNA, lipids, proteins, and other molecules in a process known as oxidative damage (1, 24, 27). The generation of ROS is also related to signaling physiological processes, such as synaptic plasticity and memory (28). C, complex; Cyt c, cytocrome c; e-, electron; ECT, electron transport chain; FAD, flavin adenine dinucleotide; Q, coenzyme Q; NAD, nicotinamide adenine dinucleotide.

Mitochondria contain two membranes, an outer and an inner membrane, an intermembrane space, and an intracellular matrix. The intracellular matrix contains several enzymes, which participate in the tricarboxylic acid (TCA) cycle and are responsible for the generation of NADH and FADH_2_ (20). These redox cofactors are required for the generation of ATP through oxidative phosphorylation *via* the ETC, present within the folds on the inner mitochondrial membrane or cristae, as explained in Figure 1 (21–23).

Given their diverse roles, mitochondria possess several mechanisms to maintain a healthy and functional mitochondrial pool (29), such as neutralizing ROS by antioxidant defenses, the unfolded protein response (UPR), mitochondrial dynamics, biogenesis, and mitophagy (30).

Apart from their involvement in cellular energy production, mitochondria also play an important role in regulating the process of apoptosis through both intrinsic and extrinsic pathways (31). In normal conditions, apoptosis removes those neurons and glia that are functionally compromised or unable to make neuronal connections (15). In the intrinsic mitochondrial-mediated pathway, stimuli such as high levels of intracellular cytoplasmic Ca^2+^ or ROS, as well as the activation of proapoptotic proteins (i.e., Bcl-2 family members) in the outer mitochondrial membrane (32), trigger a cascade of processes that activate caspases. This results in cleavage of several proteins, DNA fragmentation and cell death (33, 34). In the extrinsic pathway, activation of extracellular death receptors enhances processes that alter membrane permeability, resulting in leakage of proapoptotic factors and apoptosis (34).

Ca^2+^ homeostasis is another key process in which mitochondria are involved, with Ca^2+^ a principal secondary messenger that is involved in the regulation of neurotransmission and neuroplasticity in the brain (15). The mitochondrial outer membrane is permeable to Ca^2+^, and the inner membrane contains Ca^2+^ uniporters for its inward movement, and Na^+^/Ca^2+^ and Ca^2+^/H^+^ antiporters for its outward movement (35). Moreover, mitochondria form highly specialized signaling hubs with the ER through the mitochondria-associated membranes (MAMs), allowing the regulation of lipid synthesis and rapid transmission of Ca^2+^ signals between these organelles (36).

Mitochondrial Ca^2+^ concentrations increase when cytosolic Ca^2+^ levels are high and in case of high-energy demand, and decrease when cytosolic levels are low, or the ATP/ADP ratio is high. Ca^2+^ can modulate oxidative phosphorylation machinery by different mechanisms, including direct binding, enhancing post-transcriptional modification, and also by the activity of a Ca^2+^-dependent binding protein. It also binds to complex IV and reduces ATP inhibition of this enzyme, enhancing ATP production even in situations of high ATP concentrations (24). ATP synthesis is also enhanced by stimulation of the aspartate-glutamate carriers (AGCs) and the ATP-Mg/Pi (i.e., Ca^2+^-binding mitochondrial carrier protein, SCaMC-3) transporters on the inner mitochondrial membrane. Ca^2+^ also leads to increased NADH synthesis and higher production of pyruvate (15).

However, when Ca^2+^ levels are excessive in the intracellular space or mitochondria they induce stress and excitotoxicity, ATP production is reduced (37, 38), and Ca^2+^ is extruded through the Na^+^/Ca^2+^exchanger and the mitochondrial permeability transition pore (mPTP). Impairment in the control of mitochondrial membrane permeabilization, by mPTP, has been suggested to be responsible for the mitophagy of depolarized mitochondria, induction of apoptosis, and necrosis (15). Ca^2+^ homeostasis is regulated by different proteins, enzymes, and cellular signaling networks, which may be risk pathways for mood disorders when they are altered (15).

The maintenance of a healthy mitochondrial pool is critically regulated by the dynamics and turnover of the mitochondrial population (29). At the organelle level, mitochondrial quality is sustained through the synthesis of new mitochondria, fusion and fission, and the elimination of damaged mitochondria (30, 39). The balance between fusion and fission events shapes mitochondrial networks to meet metabolic demands (40, 41).

A considerable amount of literature has demonstrated that neuronal activity regulates mitochondria and synapses (42, 43). Neurons depend on oxidative metabolism to meet their high-energy needs (10). Thus, to match the actual local needs in neurons, mitochondria constantly move along microtubes networks, changing mitochondrial trafficking, distribution, anchoring, and membrane dynamics (44).

Mitochondria also regulate synaptic plasticity by transducing some of the effects of glutamate and BDNF. BDNF expression and signaling are promoted by some environmental factors, such as physical activity and cognitive stimulation (2, 45). On the other hand, studies have shown that BDNF enhances ATP synthesis and mitochondrial respiration through several mechanisms, including increases in glucose transport, upregulation of the mitochondrial biogenesis, and respiratory coupling efficiency (46, 47). Moreover, ATP is necessary for the mobilization of synaptic vesicles to the active sites of synapse in neurons. The ATPase complex, by producing cAMP, activates PKA kinase, which allows the mobilization of synaptic vesicles (48). When ATP production is reduced, as in mood disorders, neuronal transmission is consequently impaired (15, 49, 50).

Mitochondria also play a critical role in the neurogenesis, the process of neural stem cell proliferation and differentiation into new neurons. Numerous studies have shown that the mitochondrial genome and mitochondrial proteins are required for neuronal differentiation (51–53). Moreover, accumulating evidence has indicated that the development of a mature mitochondrial network in terms of mitochondrial function and structure is necessary during the differentiation of induced pluripotent stem cells (iPSCs) (54–56).

The aim of this article is to provide a focused narrative review of the currently available evidence supporting (1) the involvement of mitochondrial dysfunction in mood disorders, (2) the effects of current therapies on mitochondria, and (3) novel targeted therapies acting on mitochondrial pathways that might be useful for the treatment of mood disorders. To this end, a literature search was conducted to identify relevant original research articles, reviews including systematic reviews and meta-analyses containing evidence regarding the role of mitochondria in mood disorders, from MEDLINE, SCOPUS, EMBASE, ClinicalTrials, ISI Web of Science and Google Scholar. Based on these reports, we provide a critical overview of the current state of the role of mitochondria in mood disorders, ranging from physiology to pathophysiology, and therapeutic strategies, as well as perspectives on future directions.

### Take-Home Message

Mitochondria are cellular organelles involved in a number of biological processes, with a key role in maintaining neuronal homeostasis. They are involved in energy production, metabolism of ROS, calcium homeostasis, apoptosis, synaptic plasticity and neurogenesis, modulating neuronal activity and preventing neuronal damage. In mood disorders, mitochondrial dysfunction leads to the impairment in cellular homeostasis with dysregulation in these mechanisms.

## Overview and Discussion

### Mitochondrial Dysfunction in Mood Disorders

As mentioned above, mitochondria are the main source for cellular energy but are also responsible for other processes that are crucial for cell functioning and survival, such as apoptosis and Ca^2+^ homeostasis (57). Impaired mitochondrial functioning may result from a number of causes, including altered expression of mitochondria-related genes, changes in the regulation of mitochondrial biogenesis, mitochondrial structural abnormalities, changes in oxidative phosphorylation and variations in metabolite levels (57). The above-discussed functions make mitochondria indispensable in several network processes, as well as they are associated with aging and a plethora of pathological conditions, such as Alzheimer's, Parkinson's, and Huntington's disease, amyotrophic lateral sclerosis, and psychiatric diseases (6, 8, 57–60). The hypothesis that mitochondrial dysfunction is associated with these conditions is supported by reports that have associated mitochondrial diseases with psychiatric symptoms, especially mood and cognition (1, 61, 62).

### Mitochondrial Bioenergetics and Redox in Mood Disorders

The brain is an organ with the highest energy consumption, unique membrane lipid composition, and depends on mitochondrial oxidative phosphorylation, being unable to store glycogen. Since brain mitochondria produce high quantities of ATP but also ROS and RNS, this organ is vulnerable to oxidative damage, which occurs when the oxidative load exceeds antioxidant capacity (2).

#### Metabolic Changes

A number of studies using neuroimaging and post-mortem brain tissue from patients with BD have shown lower numbers of neuronal and glial cells and lower brain volume in prefrontal and limbic brain regions. Growing evidence suggests mitochondrial dysfunction is implicated in these changes through a reduction in oxidative bioenergetic generation and a shift to anaerobic glycolysis and consequently impaired neuroplasticity, phospholipid metabolism and Ca^2+^ homeostasis (15, 24). In addition, alterations in various regions of the brain in neurometabolites, including high-energy compounds, have been found in patients with mood disorders. In summary, it has been described that patients with mood disorders have lower levels of phosphocreatine (PCr), N-acetyl-aspartate (NAA), adenosine diphosphate (ADP), and ATP (63, 64). In patients with major depressive disorder (MDD), hypermetabolism could be a consequence of depression severity (65), whereas hypometabolism appears linked to less severe illness (66–69).

Moreover, studies have noted negative correlations between NAA/Creatine + PCr or NAA levels and illness duration in BD (70, 71), with an enzymatic reaction rate abnormality present in BD in the creatine kinase (CK) system, based on the decrease in the forward rate constant of the CK enzyme without alterations on ATP and PCr levels, as well as by downregulation of CK in post-mortem brains of BD patients (72, 73). This hypothesis is consistent with a previous study that showed that individuals with BD could maintain average brain concentrations of high-energy compounds at rest, but there is an underlying abnormality in the mechanism that generates new ATP, which can be uncovered when energy demand is increased (72). Apart from this, studies showing increased lactate and taurine levels and a reduced brain intracellular pH suggest that there is a shift from oxidative phosphorylation to glycolysis as a major source of energy generation in BD (74, 75). Elevated lactate is present, especially in manic phases, in the frontal cortex, caudate, cingulate, and anterior cingulate cortex, which could mean either an overall increase in ATP demand, or defective oxidative metabolism (76).

#### Mitochondrial Changes in the Electron Transport Chain

By drawing on the hypothesis of mitochondrial impairment on mood disorders, several studies in post-mortem brain, skeletal muscle or blood from patients with mood disorders have shown changes in the enzymatic activities linked to the TCA cycle and ETC, as well as impairment in mitochondrial oxygen consumption. Studies in post-mortem samples of patients with BD and MDD have shown that many mitochondria-related genes are downregulated compared with controls (77). For instance, some studies reported decreased expression of some of the complex I subunits in the cerebellum in bipolar and depressed patients compared with controls (57, 78–81). Not only is decreased expression present, but decreased activity is also reported in MDD and BD patients. A recent study confirmed previous findings showing that the citrate synthase (CS), complex II, and complex IV activities were decreased, while the complex I activity and complex I/citrate synthase ratio were significantly increased in blood platelets of BD patients during a depressive episode. Supporting these findings, Valvassori et al. (82) in isolated mitochondria from peripheral blood mononuclear cells (PBMCs) showed a decrease only in complex II activity in bipolar depressed patients. In contrast, in MDD patients, physiological respiration, the maximal capacity of the electron transport system, and respiratory rate after complex I inhibition are decreased, as well as activity of complex II and CS (83). However, there are studies on mitochondria isolated from PBMCs and blood platelet showing no significant differences in ETC activity in MDD and BD patients (84–87).

#### Oxidative Damage

Based on the premise that mitochondria are the primary source of ROS, replicated studies documented alterations in multiple aspects of oxidative stress, including an increase in the production of ROS and a reduction of the antioxidant capacity, in MDD and BD patients. Compared to healthy controls, depressed patients show an increase in oxidative stress markers involved in lipid peroxidation (88–90) and a decrease in antioxidant markers (91), as well as lower brain ATP levels (92). Patients with BD have increased lipid peroxidation products in the cingulate cortex (93), and also increased markers of oxidative and nitrosative damage in the prefrontal cortex (1, 94, 95). A meta-analysis that assessed eight oxidative stress markers in patients with BD, including 971 patients with BD and 886 controls, reported an increase of markers of lipid peroxidation, DNA/RNA damage and nitric oxide in the group with BD (96).

Since oxidative damage is the result of the balance between oxidative products and the antioxidant defense, some studies in mood disorders have investigated this system, including superoxide dismutase (SOD), catalase, glutathione S-transferase (GST), and glutathione peroxidase (GPx) (34, 97). Animal studies have shown that chronic stress is associated with lower brain concentrations of GSH, SOD and catalase (98–100). Studies in post-mortem brains of patients with BD have shown lower expression of SOD, microsomal GST, and GPx in frontal areas and lower expression of GPx in the hippocampus (101–103), and reduced activity of SOD and catalase in these patients (104, 105).

However, in some studies SOD activity appears increased in BD during the manic and depressive episodes (104, 106–108), whereas there are studies showing decreased SOD levels in manic patients (1, 109). Savas et al. (106) found increased SOD levels in euthymic bipolar patients (106), whereas others found decreased activity in the euthymic phase (104, 107). A study reported increased activity of GPx in euthymic bipolar patients (107) but not in depressed or manic patients, whereas another showed increased GPx levels in depressed bipolar patients compared to healthy controls (110). Other studies did not find any differences in GPx activity compared to a control group or across different mood states (95, 104). The same uncertain pattern is observed regarding catalase activity. Studies in chronic patients have shown decreased or unaltered catalase activity (105, 107). Contrary to these reports, BD depression at baseline presented a significant increase in catalase levels, with a lower SOD/CAT ratio (110), which was confirmed by previous findings (108). This may be explained by a compensatory mechanism in the early phases of BD, or heterogeneity in other data domains. Compared to controls, reduced GSH and glutathione S-transferase were increased among patients with late-stage BD (95).

#### Calcium-Dependent Functions

When ATP production is reduced, mitochondrial and cellular functions are impaired due to changes in mitochondrial membrane potential, reducing mitochondrial capability for Ca^2+^ uptake. Studies in brains from bipolar subjects have shown altered intracellular free Ca^2+^ levels in blood cells and olfactory neurons (111, 112). Bipolar patients evidence high cellular Ca^2+^ levels in all states, but especially in mania (113, 114), and also changes in the expression of genes involved in Ca^2+^ signaling, neuroactive ligand-receptor interaction, and protein kinase PKA/PKC signaling pathways. Moreover, the authors also found changes in the action potential system (115). Indeed, excess Ca^2+^ affects both neuronal excitability and signaling cascades regulating gene expression, leading to perturbation of multiple neuronal processes, such as dendrite development, synaptic plasticity, and excitatory/inhibitory balance (116).

Calcium/Calmodulin Dependent Protein Kinase Kinase 2 (CaMKK2), is the core component of the Ca^2+^-calmodulin (Ca^2+^-CaM) dependent signaling pathway in neurons (117). Through activation of AMP-activated protein kinase (AMPK) and the master mitochondrial regulator, PGC1α, tightly linked to the circadian clock (118), CaMKK2 regulates mitochondrial function and whole-body energy balance. Bipolar disorder is associated with mutations that affect the function or experssion of CaMKK2 (119). Decreased CaMKK2 function leads to decreased BDNF expression, a known biomarker of BD. Lastly, the activity of CAMKK2 is regulated at least in part by the multi-site phosphorylation of the catalytic domain termed the S3-node in a switchable bidirectional manner, a phenomenon critical for understanding the biphasic nature of the disorder (120, 121).

Studies have also reported that DISC1, a protein involved in mitochondrial dynamics and a putative risk factor for BD and MDD (122), interacts with the IP3R1 modulating endoplasmic reticulum-mitochondria Ca^2+^ transfer (123). One study by Dwivedi et al. (124) showed an increase in IP3R1 binding sites and protein levels in platelets of depressed patients. Moreover, Scaini et al. (125) found that BD patients had higher levels of VDAC and TSPO, suggesting that these could deregulate mitochondrial Ca^2+^ signaling and increase ROS production.

#### Mitochondrial Morphology

Other findings that support the hypothesis about the association between mitochondrial dysfunction and mood disorders are changes in mitochondrial morphology, distribution, and degradation. A study undertaken by Cataldo et al. (126) showed that prefrontal neurons from post-mortem brain samples obtained from patients with BD and peripheral cells from patients with BD contain a larger number of smaller-sized mitochondria. The same authors showed an abnormal pattern of clumping and marginalization in the intracellular distribution of mitochondria in peripheral cells, as well as atypically shaped mitochondria (ring- or cup-shaped mitochondrial profiles), suggesting subtle changes in the critical network architecture of mitochondria in the cells (127). Moreover, Mertens et al. (115) showed that iPSC-derived hippocampal dentate gyrus-like neurons of patients with BD had smaller mitochondria than those from healthy controls. As previously described, the balance of fusion and fission modifies the overall morphology of the mitochondrial network (40, 127). Thus, the alterations in these processes observed by downregulation of the mitochondrial fusion-related proteins Mfn-2 and Opa-1 and an upregulation of the fission protein Fis-1 in PBMCs from BD patients (128) might explain the abnormal mitochondrial morphology and distribution findings in patients with BD.

#### Mitochondrial Degradation and Apoptosis

By drawing on the concept of mitochondrial quality control, Scaini et al. (125) have been able to show that BD patients presented a downregulation of mitophagy-related proteins, Parkin, p62/SQSTM1 and LC3A in PBMCs, followed by NLRP3-inflammasome activation. In summary, the imbalance in mitochondrial fission and fusion toward fission, followed by a decrease in the levels of mitophagy proteins and an increase in the caspase-3 protein levels (125, 128) could suggest that the number of damaged mitochondria exceeds the capacity of mitophagy, and apoptosis becomes the dominant pathway to minimize tissue damage in BD (129, 130). Indeed, evidence has shown that apoptotic genes, such as FAS, BAK and APAF-1 are upregulated in the hippocampus of patients with BD (103). Moreover, Bcl-2, an antiapoptotic protein, is downregulated in BD patients due to different polymorphisms, resulting in Ca^2+^ homeostasis dysregulation and increased glutamate levels. This is added to the endoplasmic reticulum (ER) stress response seen in all states of BD, mainly in mania (34, 129). Chronic mild stress was shown to reduce the expression of BAG-1, a gene that enhances the anti-apoptotic effects of Bcl-2. This causes the activation of caspases, BCL-2-associated X protein (BAX), and BCL-2 antagonist/killer (BAK) in the mitochondria, which leads to the alteration of membrane permeability and neuronal death (15).

PI3K and Akt are other proteins related to cell survival and proliferation. Their transcription is upregulated in mania, and this pathway is activated by oxidative stress and IL-6, and regulated by AMPK, suggesting that this pathway is active in bipolar mania (103). Akt promotes mitochondrial survival *via* different routes, such as inhibiting cytochrome c release into the cytosol, which is the final act of mitochondrial apoptosis (131). It also activates the ETC and promotes a shift to glycolytic energy generation in BD. PI3K activates mTOR, which stimulates oxidative phosphorylation (76). Moreover, GSK-3α and GSK-3β are activated in an environment of chronic oxidative stress, such as in BD, with greater activation in mania than in depression. Their inactivation has been correlated with measured clinical improvement (76).

GSK-3 promotes cellular apoptosis by the activation of Fas receptor, also promoted by TNF-α, but which also has a role in neuroprotection. In mania, TNF-α activates GSK-3 to promote neuronal survival, since GSK-3 upregulates NFκβ, and this inhibits TNF**-**α mediated apoptosis, may inhibit oxidative phosphorylation and promote aerobic glycolysis. TNF**-**α inhibits mitochondrial biogenesis, which is prevented when SIRT-1 activity is increased (76). Increased levels of NFκβ and SIRT-1 have been found in mania compared to bipolar depression and healthy controls (76). SIRT-1 levels are lower in bipolar depression than in euthymia, and TNF-α levels may be lower in depression than in mania (76). A dysregulated NFκβ system plus genetically influenced anti-apoptotic elements might enable the increased mitochondrial function in mania and the cyclical nature of BD (132). A recent study found an association between the downregulation of 20 genes related to the apoptosis pathway, TNF-α, TLR, and NFκβ signaling pathways and major depressive disorder (76). Moreover, NFkβ causes an increase in cytoplasmic CREB levels in BD patients, which is of interest as the activity of BDNF against ROS is mediated *via* CREB transcription, and BDNF levels are lower in mania than in depression and lower in BD patients compared to controls (133). Studies also demonstrated that CREB is involved in neurogenesis and is reduced in depression (134).

#### Inflammatory Changes

Chronic inflammation has been found to be present in all phases of BD (135), since it promotes a pro-inflammatory environment with an increase in cytokine levels, such as IL-1β, IL-6, and TNF-α, and increased nitric oxide in brain and plasma (2). These changes are higher in bipolar depression than in unipolar depression and highest in mania. A meta-analysis showed that patients with major depressive disorder had higher levels of plasma IL-6, TNF-α, and soluble interleukin-2 receptors (sIL-2R) (2).

**Figure 2:**
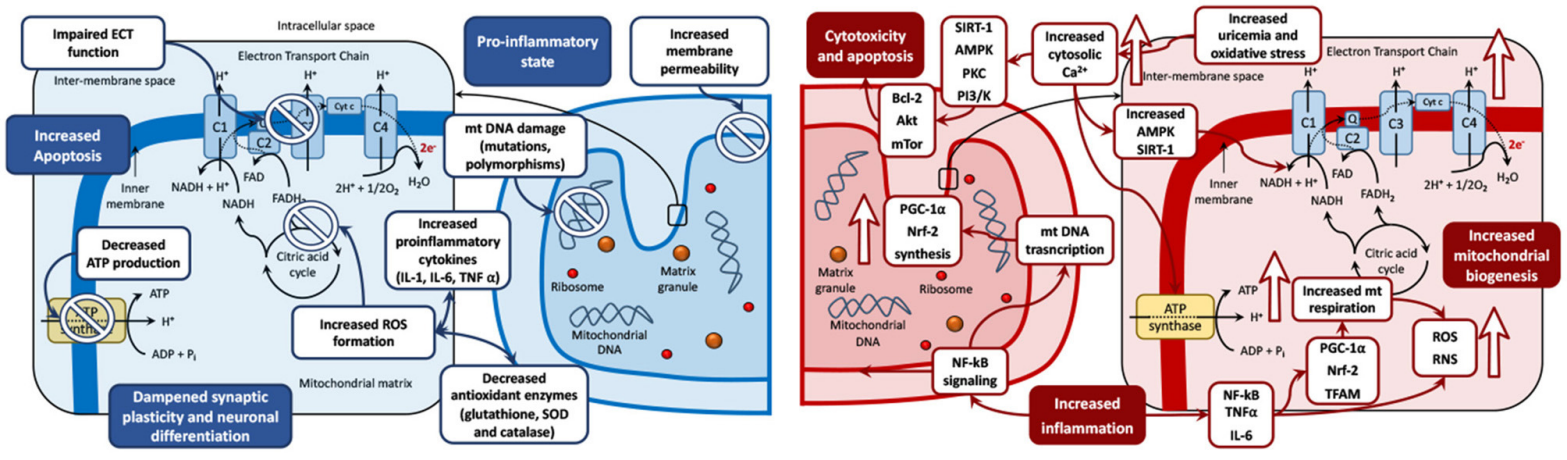
The biphasic mitochondrial model in bipolar disorders. Left: Depression mitochondrial model—Decreased mitochondrial biogenesis. Changes during depressive phases include mitochondrial DNA damage (including mutations and polymorphisms), membrane permeability, and increased formation of ROS. These imbalances lead to a pro-inflammatory state, with increased levels of pro-inflammatory cytokines (IL-1, IL-6 and TNFα) and decreased antioxidant enzymes (glutathione, SOD and catalase). These disturbances can cause cytotoxicity, increased apoptosis, and dampened synaptic plasticity and neuronal differentiation. Antidepressant drugs have shown the capacity to restore mitochondrial disregulation by reestablishing the oxidant/antioxidant balance and counteract the negative effects of depression on the mitochondria. Right: Mania mitochondrial model—Increased mitochondrial biogenesis. Upward arrows symbolize an increase. Changes during manic phases include increased inflammation and elevated production of ROS and RNS, driven by increased activity of the NF-kB signaling pathway. NF-kB signaling stimulates mitochondrial biogenesis *via* the upregulation of PGC-1α, Nrf-2, and TFAM. PGC-1α and Nrf-2 stimulate mitochondrial respiration, which is a further source of oxidative stress *via* ROS and RNS production. Increased oxidative stress could induce an increase in the levels of cytosolic Ca^2+^ ions seen in mania compared to other phases of the disease. Elevated Ca^2+^ levels can stimulate oxidative phosphorylation and ATP production and may lead to the activation of AMPK and SIRT1, which may increase the activity of NAD+. In an environment of increasing oxidative stress, the activity of SIRT1, AMPK, PKC PI3/K are increased. This can foster mitochondrial survival leading to cytotoxicity and cell death *via* activation of proapoptotic pathway cascades (Bcl-2, Akt and mTor among others). Increased uric acid levels increase the uptake of Ca^2+^ ions by mitochondria, increase the mitochondrial membrane potential and therefore enhance ATP production. Antimanic drugs including mood stabilizers and antipsychotics may restore mitochondrial dysregulation by counteracting the mitochondrial imbalance leading to neurogenesis, neuroplasticity, and cell survival. ADP, adenosine diphosphate; ATP, adenosine triphosphate; BDNF, brain-derived neurotrophic factor; C, complex; Cyt c, cytochrome c; e-, electron; ECT, electron transport chain; FAD, flavin adenine dinucleotide; IL, Interleukin; Q, coenzyme Q; mt DNA, mitochondrial DNA; mTOR, mechanistic target of rapamycin; NAD, nicotinamide adenine dinucleotide; NF, nuclear factor; Pi, inorganic phosphate; PI3/K, Inositol 1,4,5 triphosphate; PKC, Protein Kinase C; ROS, reactive oxygen species; RNS, reactive nitrogen species; TFAM, mitochondrial transcription factor A; TNFα, tumor necrosis factor-alpha.

The aforementioned evidence on mitochondrial bioenergetics pathophysiology in mood disorders is summarized in Figure 2, which represents the biphasic mitochondrial model in BD in depression (reduced mitochondrial biogenesis) and mania (increased mitochondrial biogenesis), and the derived biological processes in the mitochondria, including oxidative stress, inflammation, genetic damage, increased permeability, cytotoxicity, and apoptosis.

Other specific changes have been observed in mood disorders, which are mentioned in the next sections.

### Genetic Changes

Genetic findings also support mitochondrial dysfunction in BD. Some studies have shown that subjects suffering from mitochondrial diseases frequently develop psychiatric symptoms, especially mood symptoms (134).

Increased expression of mitochondrial fission genes and a decreased expression of mitochondrial fusion genes have been associated with depressive behavior in mice (126, 136). Pathological isoforms of DISC1 lead to abnormal neuronal development and mood disorders (137). Genome-wide association studies (GWASs) studies have identified multiple *loci*, with a small effect, associated with BD risk, including CACNA1C, ANK3, ODZ4, SYNE1, and TRANK1 (34, 122, 138, 139). In addition, Kataoka et al. (140, 141) demonstrated the potential roles of *de novo* protein-altering mutations and calcium-related genes in BD. Considering the relationship between *de novo* mutations and clinical phenotypes, the same authors observed significantly earlier disease onset among the BD probands with *de novo* protein-altering mutations when compared with non-carriers. Although no specific mutations in mtDNA have been associated with BD (142), some mtDNA haplogroups showed significantly lower cerebellar pH, which is also seen in the disorder. Moreover, a rare gene variant of mtDNA, 3644T>C, might be associated with BD, since patients showed a prevalence of 1.43% of the gene variant whereas the prevalence was 0.13% in healthy controls (34, 143). On the other hand, deletions of mtDNA were more commonly found in post-mortem cerebral cortex of patients with BD compared to controls (34, 144), and also in a patient who suffered from depression (145–147). However, other studies did not replicate these findings, which may be due to different methodologies and different brain regions studied (148).

Another study reported higher levels of circulating cell-free mtDNA in patients with MDD compared to healthy controls, while mtDNA content was not significantly different (149–151). Moreover, a recent study found a higher mtDNA copy number and a decreased DNA methylation status in the peroxisome proliferator-activated receptor-gamma coactivator 1-alpha (PGC1α) promoter in patients with MDD, which leads to reduced expression of mitochondrial genes (2, 152). In contrast, Czarny et al. (153) showed that the cellular mtDNA copy number did not differ between healthy and depressed subjects, but it showed a lower capacity for degradation and a higher number of lesions compared to controls (154).

In BD, a meta-analysis for BD-mtDNA copy number studies with a low level of heterogeneity revealed a significant lower mtDNA copy number in patients (154). In contrast, another meta-analysis with a higher level of heterogeneity identified no significant differences between mtDNA copy numbers in BD patients. A recent study showed a decrease in mtDNA copy number and an epigenetic aging acceleration in post-mortem hippocampus from BD patients (155). Focusing on mood-specific states, Wang et al. (156) found that during the depressed and manic states, patients with BD had significantly lower mtDNA copy numbers (157), with the degree of DNA damage associated with the severity of manic and depressive symptoms (157).

### Purinergic Dysfunction

The purinergic system appears dysregulated in patients with depression and BD (158). In oxidative stress, the activity of SIRT-1, AMPK, PKA, PKC, GSK, and inositol triphosphate are increased, as well as antiapoptotic proteins, such as Bcl-2, PI3K, mTOR, Akt, and uric acid. Their activation stimulates oxidative phosphorylation *via* different routes. As the mitochondrial function is increased, oxidative stress is higher and different pathways are activated in order to mitigate the cytotoxic effects of oxidative stress without inducing apoptosis.

Uric acid levels seem to be increased in all phases of BD but are higher in mania than depression or euthymia, which reflects an increase in energy production (159). Increased uric acid levels allow a greater uptake of Ca^2+^ ions by mitochondria, increased mitochondrial membrane potential, and thus higher ATP production. Lowered levels of uric acid have been described as a risk factor for developing mood disorders. Uric acid acts as a scavenger of peroxynitrite, which has high mitotoxic activity (2). It has other neuroprotective effects, such as increasing AMPK activity, which regulates the function of the CLOCK:BMAL-1 complex and upregulates the activity of SIRT**-**1, leading also to adaptative responses to oxidative stress for mitochondrial survival and functioning.

Other studies have found that cAMP and PKA are upregulated in BD and regulate the rate of oxidative phosphorylation through the phosphorylation of proteins and enzymes involved in ATP synthesis, such as cytochrome c oxidase, enhancing mitochondrial protection. Cytochrome c oxidase, the terminal respiratory enzyme, key for ATP synthesis, is a metabolic marker for neuronal functional activity (160), with its alterations related to depressive symptoms. The cAMP response element-binding protein (CREB) stimulates cAMP-dependent transcription of ETC enzyme complexes and other proteins from mtDNA, thus stimulating oxidative phosphorylation. The activity of CREB, which enhances the upregulation of CK, key for neuroprotection and energy production, is altered in BD, leading to higher or lower levels of CK in mania and lower levels in mixed states (76).

Genetic variations in the purinergic system and in a number of genes involved in cAMP signaling have been found in BD, which highlights the role of cAMP/CREB on circadian clock genes and to maintain ATP production. Higher activity of antiapoptotic proteins, enzymes and signaling cascades has been observed in mania, which enhances mitochondrial activity (2, 76).

### Circadian Clock Genes and Oxidative Phosphorylation

Oxidative stress can enhance changes in circadian clock systems, although chronic oxidative stress provokes pro-survival effects. High levels of ROS resets circadian clocks and induces a range of prosurvival responses and different expression of clock genes secondarily to a pro-inflammatory environment, such as activation of cAMP/CREB signaling. Polymorphisms in clock genes can modify cellular sensitivity to oxidative stress or genotoxic insults. Dysregulation of systems involved in oxidative stress and genetic changes in clock proteins could explain some of the observations in circadian systems in BD.

PKC and inositol triphosphate play a role in the pathogenesis of BD, being associated with and downstream of intracellular Ca^2+^ levels. In mania, elevated functioning of PKC has been found, which acts by stimulating and protecting mitochondria. Cytosolic Ca^2+^ activates ATP synthesis enhancing the activity of AMPK through different routes, such as increasing NAD+ and the activity of SIRT-1 (2).

A number of gene variations have shown increased susceptibility for developing more severe forms of BD. These genes control circadian NAD+ concentrations, which increase the activity of SIRT-1 and SIRT-3, and this stimulates oxidative phosphorylation. NAD+ and SIRT-1 directly activate ATP production and upregulate circadian genes, suggesting a pathway of influence in mood disorders (2).

### Hypothalamic-Pituitary-Adrenal Axis

Depression is linked to hyperactivity of the hypothalamic-pituitary-adrenal (HPA) axis due to an impairment of the corticosteroid receptor-mediated feedback. This leads to increased secretion of corticotropin-releasing factor (CRF) in the hypothalamus and causes increased levels of glucocorticoids both in the brain and peripherally, being translated into increased mitochondrial activity (2).

In the mitochondria, glucocorticoids form a complex with the antiapoptotic protein Bcl-2 to inhibit the formation of Bax-containing pores on the mitochondrial outer membrane. They also reduce the release of Ca^2+^ and cytochrome c from the mitochondria, inhibiting apoptosis (134, 161). Nevertheless, a chronic increase in glucocorticoid levels can cause neuronal toxicity and respiratory chain dysfunction, excessive ROS generation, apoptosis, and cell death in skeletal muscle cells and hippocampus (134, 162). Studies in rats treated with lipopolysaccharide have found sex-specific alterations in glucocorticoid receptors, which could be explained by changes in inflammation-induced expression of genes involved in oxidative metabolism (15, 163, 164).

### Glutamate and Dopamine in Mitochondrial Dysfunction

#### Glutamate

Glutamate is implicated in mood disorders. Ketamine, an N-methyl-D-aspartate (NMDA) receptor antagonist, causes a rapid antidepressant effect in patients with MDD (165, 166). This effect might be due to increased BDNF expression (134, 163), modulation by 5-HT receptors, and interactions with inflammatory processes (134, 164). Glutamate levels are higher in brains of patients with mania than other phases of illness, suggestive of dysfunction of the glutamatergic system. In depression, astrocyte density is reduced and, as a consequence, the ratio of glutamine to glutamate is not properly maintained by the glutamate recycling pathway (134, 167). Moreover, mitochondrial energy production is reduced in glutamatergic neurons in patients with MDD (168). High glutamate levels and consequently high intracellular Ca^2+^ levels promote apoptosis. On the other hand, activation of glutamate receptors also stimulates ATP production, and expression of p53, which can produce an increase of mitochondrial respiration, production of ROS and reduction of GSH (169).

#### Dopamine

Increased dopamine levels have been noted in mania. Some studies report lower dopamine transporter (DAT) binding in the striatum in unmedicated depressed or euthymic bipolar patients (2). Higher dopamine transmission and impaired DAT function in mania could be explained by elevated oxidative and nitrosative stress, which is higher in mania than in other phases of illness (170). Excessive dopamine levels in mania can also cause damage to nuclear and mtDNA by chronic nitrosative and oxidative stress. However, this is repaired by high dopamine and uric acid levels, which act in a synergistic way to repair free radical-mediated damage (76). In this environment, dopamine can protect neurons against glutamate-induced excitotoxicity, stimulate oxidative phosphorylation, and activate p53, which induces anti-apoptotic activity and inactivates tyrosine hydroxylase, which is necessary for the synthesis of dopamine. Consequently, high dopamine and glutamate levels together with high uric acid levels may not have the expected detrimental effects. Moreover, pro-apoptotic signals may induce the expression of anti-apoptotic genes such as BCL-2, inhibiting the protein Bcl-2 toxicity and apoptosis and stimulating oxidative phosphorylation (76).

All this evidence suggests that changes in mitochondrial function in MDD and BD could be key elements in order to better understand the role of the currently used pharmacotherapy and also to develop novel therapies and new treatment strategies, which will be covered in the next section.

##### Take-Home Message

Mitochondrial dysfunction may result from different causes, being some of the alterations related with several network processes in which mitochondria are indispensable.

Some of the changes observed in mood disorders include alterations in mitochondrial neurometabolites and metabolic dysfunction, decreased expression and activity of the ETC complexes, increased oxidative damage, altered calcium homeostasis, and changes in mitochondrial morphology, distribution, and degradation. In addition, increased apoptosis, chronic inflammation, Increased expression of mitochondrial fission genes and other genetic changes, including polymorphisms in clock genes, have been observed in mood disorders. Increased mtDNA degradation, purinergic dysfunction, and hyperactivity of the HPA axis, with higher glucocorticoid levels, are other findings reported in mood disorders. Finally, increased glutamate and dopamine levels have been reported in manic episodes. Nevertheless, current evidence is scarce and further studies are needed to assess these changes in mood disorders.

## Effects of Pharmacotherapy on Mitochondrial Functions

Since mitochondrial dysfunction has been related to the pathophysiology of mood disorders, including factors such as increased oxidative stress, decreased ATP production, and dysregulation of Ca^2+^ homeostasis (2), numerous studies have focused on their role as possible drug targets for pharmacological treatments (171, 172). In this regard, conventional psychotropic drugs for mood disorders, including mood stabilizers, antidepressants, and antipsychotics, have demonstrated to have molecular mitochondrial properties, such as neuroprotection, enhancement of mitochondrial function or prevention of cellular apoptosis (173), illustrated in Table 1. In addition, novel interventions are being studied and developed to be used as adjunctive therapies for mood disorders, as noted in Table 2.

**Table 1:** Effects of conventional pharmacotherapy on mitochondrial functions.

	**Molecular mitochondrial properties**	**Clinical properties**	
	**Neuronal survival**	**Inflammation and oxidative/nitrosative stress**	
**Mood stabilizers**			
Lithium (174–177)	Reduces apoptosis^*^	Prevents excessive mitochondrial calcium influx^*^	Mood-stabilizing properties in BD and antidepressant properties in MDD
	Enhanced neuroprotection and neurotrophism	Reduces oxidative stress^*^	
	Reduced cortical atrophy in BD	Antioxidant effect^*^	
Valproic acid (178, 179)	Reduces apoptosis^*^	Reduces oxidative stress in mitochondria^*^	Mood-stabilizing properties in BD
		Antioxidant effect^*^	
Antidepressants (99, 180, 181)	Reduce apoptosis^*^	Increase mitochondrial biogenesis^*^	Antidepressant properties in BD
	Enhanced neurotrophism	Reduce oxidative stress (mitochondrial and peripheral)^*^	Risk of manic switch
Antipsychotics (182–185)		Reduce oxidative stress in brain mitochondria^*^	Antimanic and mood-stabilizing properties in BD

*All data represents human clinical studies unless explicitly stated in table (*

* *animal studies)*.

*BD, bipolar disorder; MDD, major depressive disorder*.

**Table 2:** Effects of novel therapies on mitochondrial function.

**Novel therapies**	**Molecular mitochondrial properties**		**Clinical properties**
	**Neuronal survival**	**Inflammation and oxidative/nitrosative stress**	
Pramipexole (186, 187)			Antidepressant efficacy in treatment-resistant BD
**Nutraceuticals**			
N-acetylcysteine (188–190)		Reduces oxidative stress (in brain and periphery)^*^	Improves depressive and reduces manic symptoms
Omega-3 fatty acids (191, 192)		Reduce oxidative stress	Better functioning in BD
		Increase antioxidants	Improve depressive symptoms
Alpha-lipoic acid (193–196)	Reduces apoptosis^*^	Reduces oxidative stress^*^	Reverses and prevents amphetamine-induced behavioral and neurochemical alterations^*^
	Enhanced neuroprotection^*^		
Acetyl-L-carnitine (194–196)	Reduces apoptosis^*^		Improvements in depressive disorders
	Enhanced neuroprotection^*^		
S-Adenosylmethionine (197–199)		Reduces oxidative stress^*^	Improvements if supplemented in depressive disorders
			Potential risk of manic switch in BD (one study)
Creatine monohydrate (200)			Improvements in depressive symptoms
			Potential risk of manic switch in BD (one study)
Leucine, isoleucine, and valine (201)			Reduction in manic severity (one study)
L-tryptophan (202)			Reduction of manic symptoms
			Potential risk of depressive switch in BD (one study)
Carnosine (203, 204)		Reduces oxidative stress^*^	Improvement of behavior, cognition, and overall well-being
Inositol (205, 206)			Improvements in depressive symptoms in BD
Coenzyme Q10 (207)		Reduces oxidative stress	Improvements in depressive symptoms and functioning in BD
Melatonin (208–210)		Increases BDNF and ERK1/2^*^	Improvements in depressive symptoms. Scarce effects proven in BD.
		Reduces peripheral oxidative stress^*^	
Vitamin C and E (211)			Improve severity in depression
Vitamin B3 (211)		Reduces oxidative stress^*^	Enhances social behavior^*^
Folic acid (212)		Reduces oxidative stress^*^	Reduction in manic symptoms
Ketogenic diet (213)			Reports on mood stabilization

*All data represents human clinical studies unless explicitly stated in table (*

* *animal studies)*.

*BD, bipolar disorder; BDNF, brain-derived neurotrophic factor; ERK1/2, extracellular signal regulated kinases*.

### Mood-Stabilizing Drugs

Mood stabilizers are considered first-line drugs in BD to either treat mood episodes or to prevent future recurrences (214). Although the mechanism of action of mood-stabilizing drugs is not clear, some studies suggest that mitochondrial dysfunction and oxidative stress may be therapeutic targets of these drugs.

Some studies have reported that mood stabilizers, apart from altering glutamatergic neurotransmission, decrease intracellular pH, increase expression of the anti-apoptotic gene BCL-2 (by blocking the inhibition produced by histones), regulate expression of other genes, reduce elevated intracellular Ca^2+^ and increase Ca^2+^ storage capacity in the ER, and also induce mitochondrial migration to synaptic terminals, modulating neurotransmission (174, 215–217). These findings in aggregate suggest that they may reduce the symptoms of mood disorders at least in part by augmenting mitochondrial activity (63, 214). Lithium and valproate inhibit glutamate-induced apoptosis and oxidative damage to lipid and protein in cerebral cortical cells (218, 219). They also inhibit cytochrome c release from mitochondria. This reduces oxidative stress by stabilizing the inner transmembrane potential of mitochondria and prevents caspase-2 and caspase-3 activation, and cell death (174). Chronic treatment with lithium and valproate has also been shown to inhibit amphetamine-induced hyperactivity (24, 219). Lithium, valproate and carbamazepine are known to reduce inositol levels (220) and augment autophagy in cell cultures (221).

Other animal studies on the antioxidative properties of mood stabilizers have shown that chronic treatment with valproate or lithium is associated with increased ER stress proteins and related proteins, such as calreticulin (222), in cortical and PC12 cells (215, 223). These proteins are involved in antioxidative effects and mitochondrial functioning (216, 224). Moreover, lithium and valproate have other neuroprotective functions, such as regulation of the expression of GST isoenzymes in cerebral cortex, which is a group of detoxification enzymes that inhibit oxidative damage to lipid and protein in cerebral cortical cells, and GSH levels, accelerating conjugation processes (225, 226). However, with low levels of the rate-limiting synthesis enzyme (glutamate-cysteine ligase) and low levels of GSH, lithium and valproate neuroprotective effects are inhibited, which indicates that adequate GSH levels could be important for efficacy (227, 228). Other studies have shown increased oxidative damage to lipids in patients with BD, with higher lipid peroxidation ameliorated after mood-stabilizer treatment, which supports the previous findings (24, 229). Together this evidence indicates that mood stabilizers may reduce the symptoms of BD by enhancing mitochondrial activity (24).

#### Lithium

Apart from its neuroprotective functions mentioned above, lithium ameliorates BD associated cortical atrophy and maintains cortical thickness (219). Regarding Ca^2+^ regulation, lithium prevents excessive Ca^2+^ influx triggered by the N-methyl-D-aspartate receptor. In contrast, animal studies suggest that lithium can allow an increase of Ca^2+^ concentrations by desensitizing mitochondria against Ca^2+^, preventing a further response leading to apoptosis. Lithium is also correlated with increased activity of complexes I, II and III, enhances the expression of the scavenger glutathione transferase (184), facilitates mitochondrial respiration, and has other antiapoptotic properties previously mentioned (177, 230).

In rat studies lithium has been shown to inhibit inflammatory signaling pathways related to toll-like receptor 4 (TLR4), which may reduce phosphorylation of NFkβ, reducing inflammatory gene expression and also levels of caspase-3, which might prevent neuronal apoptosis (175, 231). Other studies have shown that the c-Jun N-terminal kinase, which is known to mediate oxidative toxicity, is inhibited by lithium (184, 232). Apart from enhancing this antioxidant defense, lithium has also been reported to increase the activity of SOD (233), of GPx, and total antioxidant reactivity levels in the brain (234).

Lithium also inhibits GSK-3b, a phosphorylating kinase that inhibits the conversion of pyruvate into acetyl-CoA by pyruvate dehydrogenase and also activates BAX (235). Its mutations have been shown to alter lithium response in BD. The inhibition produced by lithium may enhance ATP production and inhibit apoptosis (235, 236). Moreover, in rat models where amphetamines were used, GSK-3b was shown to enhance dopamine activation, leading to the hypothesis that lithium may contribute to maintaining normal levels of dopamine (94).

GSK-3b and phosphoinositide signaling pathways regulate BDNF, which has a complex role in mood disorders. Nevertheless, it is thought to be a potential drug target, since neurotrophic effects of lithium have been related to the increase in hippocampal BDNF in the presence of a neurotoxic insult (235, 237). Phosphoinositol is increased in patients with BD in the central nervous system and is reduced by lithium (184), causing lower levels of myoinositol in this group of patients (238, 239). In contrast, few animal studies have shown contrary effects of lithium treatment, such as the lowering of complex II and IV activity and enhanced ROS formation (240), with reduced antioxidant levels (241). It has also been shown to enhance the activity of caspase-3, leading to apoptosis (242).

#### Valproic Acid

In rats, valproate has shown to lower amphetamine-induced citrate synthase and to inhibit succinate dehydrogenase, thought to be related to its mood-stabilizing effects (243). Valproate also protects mitochondria from ouabain-induced lipid peroxidation and superoxide formation (176, 244). It could act as a cytoprotective agent in the presence of cytotoxic factors, but alone could inhibit mitochondrial functions (245, 246).

Nevertheless, other studies have shown valproate lowers levels of some cofactors, such as creatine and CoA, involved in the uptake of long-chain fatty acids into mitochondria, which leads to reduced beta-oxidation (214). Valproate has also been shown to enhance ROS generation by inhibition of complex II, and to induce mPTP opening, with a reduction of membrane potential, leading to the release of cytochrome c and apoptosis. Valproate inhibits ATP synthesis when pyruvate is used as a substrate (214). In rat studies, valproate has shown to inhibit glutamate-driven oxidative phosphorylation (247, 248). In cases of impaired ETC structure, valproate inhibits complexes I and IV activity and SOD levels (179).

#### Other Mood Stabilizers

Although there is scant evidence regarding other mood stabilizers apart from lithium and valproate, changes found in animal studies with carbamazepine treatment include the reduction in mitochondrial respiration, ATP synthesis, and membrane potential, and also the inhibition of Ca^2+^-induced swelling of liver cells (241, 249, 250). Lamotrigine has been shown to inhibit the effects of rotenone, a cytotoxic agent, and maintain mitochondrial membrane potential, preventing mPTP opening and increasing GSH levels (251). Its neuroprotective effects could be due to complex I inhibition (252).

### Antidepressants

In animal models of depression (214, 253), antidepressants seem to increase mitochondrial biogenesis and enhance antioxidative capacity against oxidative stress (139, 184). For instance, venlafaxine increases expression of anti-apoptotic and antioxidant mitochondrial genes (254), and agomelatine may similarly scavenge free radicals (11, 255). As with electroconvulsive therapy, they reduce peripheral inflammatory cytokines (33), which is supported by the reported antidepressant activity of celecoxib, a cyclooxygenase 2 (COX-2) inhibitor (256, 257). Antidepressants also increase autophagy and neural plasticity (134, 258). Since mitochondrial dysfunction has been linked to mental disorders (184), cytochrome c oxidase and apoptosis inhibition have been studied as potential new treatment approaches (259).

In patients treated with antidepressants, there is an increase in the levels of BDNF mRNA (260) and a reversion of the decrease in CREB levels seen in patients with depression (261), which could be mechanisms of action in mood disorders (262). Animal studies have also shown that some antidepressants inhibit complex I in brain mitochondria, reducing its metabolic function (263). A number of studies have shown that some antidepressants, including fluvoxamine, fluoxetine, sertraline, paroxetine, nortriptyline, and venlafaxine, alter ETC activity in mitochondria (241, 264). The reduction of ROS production could explain their beneficial effects (265–267).

Fluoxetine also promotes cytochrome c oxidase and glutamate dehydrogenase activity in presynaptic mitochondria of rat hippocampus (160, 268), and inhibits multiple other enzymes in mitochondria (11, 181). Increased cytochrome c oxidase activity in the female hippocampus by fluoxetine could improve outcomes in women (269–271). Apart from altering mitochondrial energy production, fluoxetine might affect the mitochondrial processes *via* the glucocorticoid receptor (GR) (33, 272).

Antidepressants are also involved in apoptosis, playing a complex role that depends on cell and brain structure type. One study reported that paroxetine, fluoxetine and clomipramine increased levels of apoptotic markers (cytochrome c and DNA fragments), but imipramine did not have any effect (24, 273). Desipramine induced apoptosis by activating the caspase pathway in glioma cells (274), while fluoxetine and amitriptyline protected PC12 cells from cell death (275). Nortriptyline inhibited neuronal cell death, protecting isolated mitochondria against programmed cell death, inhibiting the release of apoptotic mitochondrial factors and caspases (276). Fluoxetine has been shown to prevent stress-induced apoptosis in the hippocampus, but not in the prefrontal cortex (277, 278). In summary, different mitochondrial functions, such as ATP synthesis, generation of ROS, and cell death, are important targets of antidepressants.

### Antipsychotics

Few studies have explored mitochondrial modulation by antipsychotics (279, 280). Olanzapine has shown to increase SOD activity and protect PC12 cells from oxidative damage by H_2_O_2_ (20, 281), and also to prevent the decrease in membrane potential and ROS overproduction induced by beta-amyloid peptide (282). In two mice studies, quetiapine increased mitochondrial ETC activity and reduced markers of oxidative stress in the prefrontal cortex, nucleus accumbens, amygdala, and hippocampus (24, 282–284). Scaini et al. [183, 185, 186) showed a significant decrease in all the functional parameters of mitochondrial oxygen consumption after treatment with clozapine and olanzapine in lymphoblastoid cell lines (LCLs) from healthy controls, and these effects were more prominent in cells treated with olanzapine. The same authors also demonstrated that the treatment with clozapine and olanzapine at high doses further decreased mRNA expression of Mfn-2 and Drp-1 in LCLs, supporting the notion that clozapine and olanzapine can potentiate mitochondrial dysfunction.

### Novel Therapies

In the last years, a number of agents have been studied as potential therapeutic factors aimed to treat and improve the course of mood disorders, including factors involved in the glutamatergic pathway, insulin transduction pathway, melatoninergic system, purinergic system, endopeptidases, and also mitochondrial modulators (183). A number of the latter agents have been developed or studied with the aim of enhancing antioxidant defenses or mitochondrial functioning as adjuvant therapy to antidepressants (285).

Studies of Ca^2+^ channel blockers, such as diltiazem and verapamil, have been conducted as potential treatments for BD, but results in the literature are still controversial. It was hypothesized that their therapeutic effect may be due to the protection of neurons against the damage induced by excessive Ca^2+^ levels (2).

Pramipexole, a D2/D3 agonist approved for the treatment of Parkinson's disease and restless legs syndrome, upregulates Bcl-2 (286). It has also shown antidepressant efficacy in treatment-resistant bipolar patients (287), with a superior response rate compared to placebo and similar to SSRIs.

Some dietary supplements (or nutraceuticals) have been assessed as potential treatments in mood disorders (186, 187), since they may enhance mitochondrial function and brain energy metabolism and prevent ROS-induced damage. These include N-acetylcysteine (NAC), alpha-lipoic acid (ALA), acetyl-L-carnitine (ALCAR), S-adenosylmethionine (SAMe), coenzyme Q10 (CoQ10), creatine monohydrate (CM), and melatonin (201).

The molecular mitochondrial properties shown by novel therapies for mood disorders are summarized in Table 2.

#### Nutraceuticals

##### N-Acetylcysteine

N-acetylcysteine (NAC) is a GSH precursor, the major antioxidant agent in the brain (288) for preventing oxidative damage in the mitochondrial ETC (289). By increasing GSH levels, NAC may increase mitochondrial respiratory capacity and have neuroprotective functions by other mechanisms (285, 290). It can prevent oxidative damage to complex I (184), can enhance GST activity, and act directly against oxidant radicals (290, 291).

Some studies in BD have demonstrated that treatment with NAC can improve depressive symptoms, clinical response rates, symptom remission, quality of life and functioning (292, 293). Few clinical trials assessing the efficacy of NAC as adjunctive treatment in patients with BD have shown promising results (189, 281, 289, 294–298), with benefits in depressive symptoms of BD patients (63, 189, 285, 289), but not as maintenance treatment (189, 294). *Post-hoc* analyses suggested that NAC might be effective in later stages of BD (289) and also to reduce manic symptoms (281). However, there are recent negative trials, albeit smaller and of a shorter duration (298).

Clinical trials in depressive disorders also suggest the potential of NAC as adjunctive treatment in depression (299, 300). Although no differences in depressive symptoms were found in another clinical trial comparing NAC with placebo (189, 301), the NAC group showed a better response at the 16-week post-discontinuation endpoint. A meta-analysis including five studies assessing depressive symptoms with a follow-up of 12–24 weeks revealed significantly greater improvements in depressive symptoms and functionality with NAC compared to placebo (190).

##### Omega-3 Fatty Acids

Some studies have demonstrated modulatory effects of omega-3 fatty acids on mitochondria. Eicosapentaenoic acid (EPA; 20:5n-3) is a fatty acid that seems to protect against oxidative stress by replenishing oxidized lipids and increasing oxygen and glucose supply to the brain (301). Diets rich in omega-3 fatty acids have shown to upregulate cytochrome c oxidase, cytochrome b, and ATP synthases, leading to increased ATP formation (302, 303).

In a study of rodents with methylphenidate-induced mania, omega-3 fatty acids alone and in combination with lithium and aripiprazole reduced levels of SOD, CAT, and lipid peroxidation products (304). Stanley et al. (63, 305) demonstrated that docosahexaenoic acid (DHA; 22:6n-3) changes mitochondrial membrane phospholipid composition and mitochondrial function, protecting mitochondria against damage (306). A pilot study found significantly higher remission, greater improvements in depressive symptoms, and better global functioning in bipolar patients supplemented with omega-3 fatty acids, while no benefits in mania were found (306). Clinical improvements associated with omega-3 fatty acids intake are produced at least in part by modulation of BDNF levels (192). A study that included 10 different countries found a correlation between lower fish or seafood consumption with a higher prevalence of bipolar spectrum disorders (307, 308). Patients with BD have shown lower levels of erythrocyte DHA, ALA and EPA when compared to healthy controls (63, 309). Another study showed a trend toward lower levels of omega-3 fatty acids in relatives of patients with BD (191).

A systematic review of clinical trials assessing nutraceuticals showed positive and statistically significant results on depression in four out of nine studies (201), but none showed positive findings in mania. However, sample sizes were small, reducing the chance of positive results (192, 310). The previous evidence suggests that supplementation or increased consumption of omega-3 fatty acids may be beneficial in mood disorders, but additional studies are necessary to define their clinical efficacy more accurately.

##### Alpha-Lipoic Acid

Alpha-lipoic acid (ALA) is an antioxidant found in red meats, spinach, yeast, and other products (201). It facilitates glucose entrance into cells for ATP synthesis and recycling of endogenous antioxidants, such as CoQ10, vitamins C and E, and GSH (193). ALA has been demonstrated to reduce metabolic deficits, oxidative stress and apoptosis (by preventing glutamate-induced Ca^2+^ cellular influx) (311, 312), stimulate glucose uptake into cells, improve cognitive function and enhance neuroprotection (312, 313) and to stimulate mitochondrial biogenesis (195), but studies in mood disorders are lacking (312).

##### Acetyl-L-Carnitine

L-carnitine (ALCAR) is a compound obtained through the diet (314) that is biosynthesised from lysine and methionine. It enhances the entrance of fatty acids into the mitochondria for ROS scavenging and beta-oxidation, which leads to ATP and acyl-coenzyme A (acyl-CoA) production (315). Acyl-CoA enters the citric acid cycle (316, 317). Reported functions of ALCAR include neuroprotection, anti-apoptotic properties (315), inhibition of GABA production (316, 318), and enhancing of mitochondrial functioning (319). Animal studies of ALCAR show increased levels of ATP and PCr (195). Results regarding the reduction of oxidative stress are mixed, but coadministration with NAC or ALA has shown benefits (320). Moreover, supplementation with ALA and ALCAR may promote mitochondrial integrity in the hippocampus of aged rats (321). Some early clinical trials suggest the ALCAR has significantly greater efficacy than placebo as an augmentation treatment depressive disorders (63, 194). However, another study found no significant differences in depressive scores of ALCAR/ALA treatment compared to placebo (322–324).

##### S-Adenosylmethionine

S-Adenosylmethionine (SAMe) is formed from ATP and methionine and is needed for the synthesis of many neurotransmitters and for repairment and degradation of dysfunctional proteins. It is a precursor for GSH production, which plays an important role in reducing oxidative stress. It is also used for homocysteine synthesis, which in turn can regenerate SAMe (196).

Some studies suggest the efficacy of SAMe supplementation for depressive episodes as adjunctive therapy with a number of antidepressants SSRI, venlafaxine, or SNRIs (288). Nevertheless, studies with older antidepressants, including phenelzine, mianserin, and maprotiline, showed inconsistent results (197, 199, 325–327). One study in patients with BD showed SAMe might pose a risk for a manic switch (288), but randomized clinical trials in BD are still lacking (328). A recent large scale trial showed a numerical but not statistically significant benefit of SAMe in depression (198).

##### Creatine Monohydrate

Creatine is an antioxidant agent synthesized by the liver and kidneys which is also found in meat and fish (288). It can be obtained as a supplement in the form of creatine monohydrate (CM). Creatine is the precursor of PCr, a reservoir of inorganic phosphate, used for ATP synthesis by donating a phosphate to ADP (329). In the context of high-energy demand, PCr is rapidly converted to creatine to donate a high-energy phosphate to ADP to obtain ATP (330).

Creatine also attenuates the decreases in N-acetyl-aspartate (NAA), which acts as a marker of impaired mitochondrial function, and inhibits the activation of the mitochondrial permeability transition, suggesting neuroprotective effects (331, 332). Other neuroprotective effects are intracellular Ca^2+^ buffering, extracellular glutamate reduction, and antioxidant effects (333–335). PCr and NAA concentrations are reduced in BD patients and this reduction correlates with clinical severity (336). Where PCr levels are diminished, CM supplementation may increase PCr and NAA production to promote neuroprotection (70, 337).

Despite limited evidence in mood disorders, CM has been associated with improvement in depressive symptoms in case studies (63). Benefits were seen in treatment-resistant depression in a small open-label study (338), which also suggested a risk of a manic switch after CM treatment in patients with BD (200). Before CM might be considered as an adjunctive treatment for the management of BD and depressive disorders, RCTs are necessary.

##### Other Aminoacids

In one study assessing the efficacy of leucine, isoleucine, and valine combination vs. placebo in 25 patients with BD, positive results were seen with significant reductions of the severity of mania within 6 h in the verum group, whose activity may be explained by competitive inhibition of phenylalanine and tyrosine, which are necessary for dopamine synthesis (288). L-tryptophan reduced manic symptoms in a study of 24 patients (339, 340). Moreover, a meta-analysis reported significantly reduced plasma tryptophan levels in patients with MDD (202). This aminoacid was shown to reduce depression scores in people with unipolar depression in methodologically limited studies, so further evidence is required in order to consider it as an adjunctive therapy (341).

Carnosine is a dipeptide made up of the amino acids beta-alanine and histidine that protects brain mitochondria and regulates the immune system (201, 342). It has also been studied for adjuvant treatment of depression (343, 344). So far, it has been demonstrated to reduce the effects of chronic stress in animal studies and to improve behavior, cognition, and overall well-being in human studies (204, 345).

##### Inositol

A pilot study using inositol, a glucose isomer, in 24 patients with BD found a significant reduction in depression scores after 3 weeks of treatment but not after 6 weeks (2, 203). Another 6-week study in 17 subjects with BD showed no significant reduction in depression or mania scores (206). Notwithstanding, both studies found a greater clinical response with inositol compared to controls, which suggests a potential benefit of this agent in BD (205).

##### Coenzyme Q10

Coenzyme Q10 is a component of the ETC complex involved in ATP synthesis (201). It acts as an antioxidant in mitochondria and lipid membranes (346). CoQ10 has been suggested to stabilize the mitochondrial membrane in the context of oxidative stress (63, 347). It also inhibits the activity of mPTP and increases complex I activity (348). One open-label placebo-controlled trial reported clinical improvement in depressive symptoms in older adults with bipolar depression using this supplement (63).

A randomized controlled trial comparing nutraceutical treatment (including ALC, CoQ10 and ALA, in addition to co-factors involved in mitochondrial function) with NAC and placebo in patients with depression did not show a significant difference between groups at the primary endpoint. However, the rate of change between baseline and week 20 post-discontinuation was significantly greater in the group previously treated with nutraceuticals compared with the placebo group on depression scores, and also on functioning. This suggests a delayed benefit of the combination or improvement of symptoms on withdrawal, which should be assessed in future studies (285). Thus, current evidence suggests that CoQ10 might be beneficial in mood disorders (288), but further clinical trials in mood disorders are necessary to confirm these early promising but non-definitive signals.

##### Melatonin

Melatonin is a hormone released in a circadian pattern by the pineal gland and other tissues in the body, including the brain. It has a number of functions and is an important antioxidant free radical scavenger (207, 288). Specifically, it stimulates the production of GSH (349) and increases the expression of genes related to antioxidative functions, such as glutathione peroxidase and SOD (210). Melatonin also seems to directly enhance mitochondrial function since it activates ETC complexes, increases mitochondrial membrane fluidity, and closes the mPTP. It also protects mtDNA against degradation, promotes the expression of mitochondrial genes coding for ETC complex subunits, and has neuroprotective properties (209). The beneficial effects of melatonin seem to be those related to ROS scavenging and actions linked to cytosolic proteins (209, 288).

Clinical studies in mood disorders do not show conclusive results. So far, melatonin has shown benefits improving depressive symptoms in patients with “winter depression” (209, 288) compared to placebo, whereas a controlled study in seasonal affective disorder did not show changes in atypical depressive symptoms (208), and a crossover study on patients with severe depression showed that patients taking melatonin had worsened dysphoria, sleeping patterns and weight gain (350).

Regarding evidence assessing melatonin for BD, one open-label study showed no significant effects on mood or sleep in rapid-cycling patients (351), whereas in another small open-label study it showed sleep-enhancing and antimanic effects in manic patients (352). As mentioned previously, agomelatine, an agonist of melatonin MT1 and MT2 receptors, has demonstrated preliminary evidence of efficacy in bipolar depression (353), but agomelatine has other actions on the serotonin system.

##### Vitamins and Minerals

Supplementation with vitamins C and E was shown to significantly improve severity in depression. One study where they were combined with monoaminergic antidepressants for 12 weeks showed they improved oxidative stress in subjects with MDD (354), but it was not a placebo-controlled design. Nicotinamide is a form of vitamin B_3_ found in food, used as an antioxidative substance, and is also a precursor of NAD+. It is hypothesized to be effective for the treatment of mood disorders (211), since it increases oxidative phosphorylation in the brain and enhances social behavior in high-anxiety rats (355). A small clinical trial evaluating magnesium as a potential adjunctive therapy for treating acute mania or rapid cycling BD showed a greater reduction of manic symptoms compared to controls (356), which might be due to the modulation of Ca^2+^ channel activity and its participation in neurotransmitter release (357). One study using folic acid for 17 BD participants showed no statistically significant differences on symptoms of depression compared to controls (201, 358), but in another where 88 patients with acute mania were initiated on valproate, folic acid at doses of 3 g showed a significant reduction of manic symptoms at week 3 compared to placebo (359). Thus, assessing folate levels and administering supplementation in patients with mood disorders could be beneficial for the clinical course of the acute episodes.

##### Ketogenic Diet

The effects of specific diets in mood disorders are still not clear despite evidence reporting that they can alter several biological processes. The exception is the Mediterranean diet, which has been associated with antioxidative properties and has shown antidepressant effects in a RCT (201, 360). One study assessing rats on a calorie-restricted diet showed that mitochondrial efficiency and oxidative damage in skeletal muscle were significantly increased in these rats, while antioxidant effects were significantly lowered in food-restricted rats that followed a high-fat diet. Thus, caloric restriction seems to predispose to higher mitochondrial efficiency and also to high-fat induced oxidative damage (361). Other studies have shown that the ketogenic diet (KD) upregulates mitochondrial antioxidant status and protects mtDNA from oxidant-induced damage (362). It has also shown effective anticonvulsant properties and has been suggested as a potential adjunctive therapy as a mood stabilizer.

The ketogenic diet consists of a low-carbohydrate diet that substantially changes the energetic source of the organism (213, 363), which switches from glucose to ketones bodies, obtained by breakdown of fatty acids. This causes alterations in neurotransmitter levels, hormones, and peptides (364), and an increase in oxidative phosphorylation and ATP synthesis (365, 366), increased GSH levels, reduced ROS production (367), reduced inflammatory levels and neuroprotection (368). A ketogenic diet seems to influence epigenetic changes involved in increased mitochondrial function and biogenesis (369), which might also be responsible for the increase of BDNF (370). The ketogenic diet stimulates the endogenous antioxidant system through the activation of nuclear factor erythroid-derived 2 (NF-E2)-related factor 2 (Nrf2), the major inducer of detoxification genes (371, 372), especially in the hippocampus (373). Despite the limited data regarding the ketogenic diet for the treatment of mood disorders, early reports support the hypothesis about its beneficial effects on mood stabilization (374).

### Physical Exercise

Physical activity is directly related to increased mitochondrial biogenesis, increased mitochondrial content and oxygen utilization capabilities, and that aerobic exercise in the elderly ameliorates loss of skeletal muscle mitochondrial content (369). One study assessing the efficacy of fluoxetine and exercise in muscle cells of rats reported that physical activity increased cytochrome c oxidase activity compared with the group treated only with fluoxetine. Exercise increased citrate synthase activity in both fluoxetine and non-fluoxetine groups, and fluoxetine increased its activity only in the exercise group. On the other hand, exercise significantly decreased ROS levels in both fluoxetine and non-fluoxetine groups, with this reduction higher in the fluoxetine group (375). *Post-hoc* analysis of a trial of a mitochondrial combination therapy found the greatest benefits in those with the highest levels of physical activity (376). There is a meta-analytic level of evidence from RCT's that exercise has antidepressant effects. Hence, enhancing mitochondrial function through physical activity may provide a novel way to treat mood disorders (377).

#### Take-Home Message

Numerous studies have focused on the role of therapeutic agents targeting different mitochondrial functions that are altered in mood disorders. On one hand, mood stabilizers, antidepressants and antipsychotics have shown to promote neuroprotection, reduce oxidative stress and enhance mitochondrial function. On the other hand, novel interventions have been assessed as potential adjunctive therapies for mood disorders.

Some mitochondrial modulators have been developed or studied with the aim of enhancing antioxidant defenses or mitochondrial functioning as adjuvant therapies in mood disorders. Pramipexole has shown antidepressant effects by the upregulation of Bcl-2. Some dietary supplements or nutraceuticals have been found to enhance mitochondrial function and brain energy metabolism mainly by the reduction of oxidative stress. These include N-acetylcysteine (NAC), alpha-lipoic acid (ALA), acetyl-L-carnitine (ALCAR), S-adenosylmethionine (SAMe), coenzyme Q10 (CoQ10), creatine monohydrate (CM), and melatonin. Even though current evidence suggests they might be beneficial in mood disorders, further clinical trials are necessary to confirm these findings.

Melatonin has antioxidative functions and also enhances mitochondrial function. However, clinical studies in mood disorders have not shown positive results. Vitamin supplementation, ketogenic diet and physical exercise have also shown positive effects in mitochondrial function and mood disorders, with scarce evidence.

## Conclusion

Mitochondria play a key role in different cellular functions, especially those related to energy production. A number of studies indicate the possible role of mitochondria in the pathophysiology of mood disorders, raising the possibility that the processes of energy generation and oxidative damage could be significant therapeutic targets for the treatment of BD with mood-stabilizing or other kinds of drugs as well as lifestyle approaches. A better knowledge of mitochondrial functioning could help identify impaired processes and specific treatment targets. This would increase the understanding of mechanisms of action of the drugs currently used and aid the development of novel effective therapies to treat specific mitochondrial functions that might be used as the main therapy or as adjunctive treatment, especially for subjects that do not fully respond to conventional therapies. Research on changes in mitochondrial processes in patients with mood disorders might clarify how mitochondrial dysfunction can be considered a biological target. Further studies are needed to confirm that pharmacological treatments reduce or delay neuroprogressive changes in mood disorders, and to demonstrate the potential benefits of putative antioxidant substances.

## Author Contributions

All authors contributed in the preparation of the manuscript and gave approval for the final version.

## Conflict of Interest

AG-P has received support from Janssen-Cilag and Otsuka-Lundbeck, and declares no support related to the subject of this article. SD has received grant support from Stanley Medical Research Institute, NHMRC, Beyond Blue, ARHRF, Simons Foundation, Geelong Medical Research Foundation, Fondation FondaMental, Eli Lilly, Glaxo SmithKline, Organon, Mayne Pharma and Servier. He has received speaker's fees from Eli Lilly, advisory board fees from Eli Lilly and Novartis and conference travel support from Servier, with no financial or other relationship relevant to the subject of this article. GA has received CME-related honoraria, or consulting fees from Janssen-Cilag, Lundbeck and Angelini with no financial or other relationship relevant to the subject of this article. JQ received clinical research support from LivaNova; has speaker bureau membership with Myriad Neuroscience, Janssen Pharmaceuticals, and Abbvie; is consultant for Eurofarma; is stockholder at Instituto de Neurociencias Dr. Joao Quevedo; and receives copyrights from Artmed Editora, Artmed Panamericana, and Elsevier/Academic Press. IP has received CME-related honoraria, or consulting fees from ADAMED, Janssen-Cilag and Lundbeck. EV has received research support from or served as consultant, adviser or speaker for AB-Biotics, Abbott, Allergan, Angelini, Dainippon Sumitomo Pharma, Ferrer, Gedeon Richter, Janssen, Lundbeck, Otsuka, Sage pharmaceuticals, Sanofi-Aventis, Shire, Sunovion, Takeda, and reports no financial or other relationship relevant to the subject of this article. MB has received Grant/Research Support from the NIH, Cooperative Research Centre, Simons Autism Foundation, Cancer Council of Victoria, Stanley Medical Research Foundation, Medical Benefits Fund, National Health and Medical Research Council, Medical Research Futures Fund, Beyond Blue, Rotary Health, A2 milk company, Meat and Livestock Board, Woolworths, Avant and the Harry Windsor Foundation, has been a speaker for Abbot, Astra Zeneca, Janssen and Janssen, Lundbeck and Merck and served as a consultant to Allergan, Astra Zeneca, Bio- advantex, Bionomics, Collaborative Medicinal Development, Janssen and Janssen, Lundbeck Merck, Pfizer and Servier, and has licences with Allen and Unwin and Cambridge University Press. MB has received patents for agents that modulate physiological processes and diseases of the central nervous system and related processes, including xanthone-rich plant extracts. All are unrelated to this work. All other authors report no financial or other relationship relevant to the subject of this article.
